# Extinction vs. Abstinence: A Review of the Molecular and Circuit Consequences of Different Post-Cocaine Experiences

**DOI:** 10.3390/ijms22116113

**Published:** 2021-06-06

**Authors:** Marek Schwendt, Lori A. Knackstedt

**Affiliations:** 1 Psychology Department, University of Florida, Gainesville, FL 32611, USA; schwendt@ufl.edu; 2 Center for Addiction Research and Education, University of Florida, Gainesville, FL 32611, USA

**Keywords:** mGlu5, mGlu1, GluA1, Glu2, Narp, PSD-95, Homer, reinstatement, context, cue

## Abstract

The intravenous cocaine self-administration model is widely used to characterize the neurobiology of cocaine seeking. When studies are aimed at understanding relapse to cocaine-seeking, a post-cocaine abstinence period is imposed, followed by “relapse” tests to assess the ability of drug-related stimuli (“primes”) to evoke the resumption of the instrumental response previously made to obtain cocaine. Here, we review the literature on the impact of post-cocaine abstinence procedures on neurobiology, finding that the prelimbic and infralimbic regions of the prefrontal cortex are recruited by extinction training, and are not part of the relapse circuitry when extinction training does not occur. Pairing cocaine infusions with discrete cues recruits the involvement of the NA, which together with the dorsal striatum, is a key part of the relapse circuit regardless of abstinence procedures. Differences in molecular adaptations in the NA core include increased expression of GluN1 and glutamate receptor signaling partners after extinction training. AMPA receptors and glutamate transporters are similarly affected by abstinence and extinction. Glutamate receptor antagonists show efficacy at reducing relapse following extinction and abstinence, with a modest increase in efficacy of compounds that restore glutamate homeostasis after extinction training. Imaging studies in humans reveal cocaine-induced adaptations that are similar to those produced after extinction training. Thus, while instrumental extinction training does not have face validity, its use does not produce adaptations distinct from human cocaine users.

## 1. Introduction

Approximately 25 million Americans report use of illicit drugs, including cocaine (National Survey on Drug Use, Rockville, MD, USA, 2013). Cocaine use disorder (CUD) has one of the highest potentials for abuse and chronic relapse. The risk for relapse persists even after months to years of cocaine abstinence (Dackis and O’Brien, 2001). To date, effective pharmacotherapies to reduce relapse remain elusive. Progress toward this goal requires understanding of the neurobiological mechanisms underlying relapse. Animal models of relapse are critical for determining such mechanisms.

The rodent model most commonly used to study relapse is the intravenous self-administration (IVSA) model. Rodents are trained to self-administer cocaine in an operant chamber, wherein an operant response (i.e., lever press or nose poke) results in the delivery of IV cocaine. Some, but not all, studies utilize cocaine-paired discrete cues such as a stimulus light and/or tone. After weeks of IVSA, rodents are put through a cocaine-free period which typically involves either simple drug abstinence in the home cage or extinction training. Extinction training involves extinguishing the association of instrumental responses and/or drug-paired cues with drug delivery. Some studies extinguish only the lever-drug association by conducting instrumental extinction in a context distinct from the self-administration chamber. Others extinguish the drug-paired cues, but not the instrumental response. The most common type of extinction used in cocaine reinstatement studies is instrumental extinction in the IVSA context. “Voluntary abstinence” models, where rodents are given a choice between drug and another reinforcer such as palatable food, have been used for methamphetamine and opioid studies, but have yet to be employed for cocaine studies [1]. A punishment model, in which foot-shock is delivered to induce voluntary abstinence from cocaine has been used in at least two studies to date. Following a drug-free period, relapse to drug-seeking is induced by the drug itself (non-contingent cocaine), drug-paired cues, the drug-paired context, or cues+context. The term “reinstatement’ refers to the resumption of an extinguished instrumental drug-seeking response after priming by drug-paired discrete cues, stress, or the drug itself. When abstinence is used in combination with a cue+context-primed relapse test, the term “incubation of cocaine craving” has been used, because the number of instrumental responses made on the previously active drug lever or port increase with the length of drug abstinence [2].

Such experimental models of drug relapse are used to screen translational compounds for the treatment of relapse, as well as to identify the neurocircuitry and molecular neuroadaptations underlying such relapse. Thus, it is necessary to use animal models with the highest possible degree of clinical translation. In recent years, attention has been given to the fine details of IVSA-based relapse models, finding that parameters such as length of daily cocaine IVSA access, intermittency of access (vs. continuous cocaine access), and polysubstance use (e.g., consuming a second drug with cocaine) influence the neurobiology of cocaine seeking [3,4,5,6,7]. The behavioral and neurobiological consequences of these factors have been the focus of other reviews (e.g., [8,9]). Here, we will focus on a relatively neglected topic: the impact of post-cocaine IVSA treatment abstinence procedures on the circuits regulating cocaine-seeking. We will first discuss differences in the neurocircuitry mediating cue- and context-primed relapse following extinction and abstinence. We will focus on these types of relapse primes so that we can better compare across abstinence regimens. The majority of the literature on stress- and cocaine-primed reinstatement utilizes instrumental extinction procedures, making it impossible to compare the effects of extinction and abstinence on the circuits mediating these types of relapses. We will then discuss molecular changes observed in a key node of the reward circuitry for cue- and context-primed relapse—the nucleus accumbens (NA). Finally, we will discuss the results of pharmacological interventions for cocaine relapse that have been tested following both extinction and abstinence.

## 2. Neurocircuitry Mediating Cocaine Relapse

Separate, but overlapping, circuitries mediate relapse to cocaine-seeking depending on whether rats experience abstinence or instrumental extinction. Additionally, playing a role is the context of such extinction (in the IVSA context or not), as well as the relapse prime (Table 1). When instrumental extinction is employed, the dorsomedial (dm) prefrontal cortex (PFC), NA core, dorsal striatum (dSTR), basolateral amygdala (BLA), ventral tegmental area (VTA), and ventral hippocampus (vHipp) are required for cue-primed reinstatement, but the ventromedial (vm) PFC is not [10,11,12,13,14]. The dmPFC is also necessary for context-primed relapse when extinction training is conducted in an alternate context to self-administration and relapse testing [13]. When rats are trained to self-administer cocaine in the presence of discrete drug-associated cues and then undergo abstinence without extinction, inactivation of the dmPFC does not attenuate cue+context-primed relapse [15]. However, infusion of ifenprodil, a specific antagonist of GluN2b-containing NMDA receptors, directly into the dmPFC attenuates this type of relapse [16]. When rats are trained in the absence of discrete cues and then undergo abstinence, the dmPFC is not necessary for context-primed relapse [12]. Conversely, chemo- or opto-genetic *activation* of the vmPFC (and its projections to the NA shell) decreases cued-primed reinstatement after 21–28 days of extinction from both short (2 h/day) and extended (6 h/day) access to cocaine self-administration [17,18]. These studies found that the same manipulation did not attenuate cued-cocaine seeking after a similar period of abstinence without extinction. However, another study used an optogenetic approach to activate the vmPFC-NA shell projection, finding that such activation reduces incubated cocaine seeking after 15 days of abstinence [19]. This study used different methods, including conducting both IVSA and cue-induced drug seeking tests using discrete trials. Activation of the vmPFC-NA shell occurred in 50% of trials, and a within-subjects comparison found that stimulation of this pathway reduced incubated cocaine seeking. This study also employed a supraphysiological stimulation of vmPFC neurons, in comparison to the stable step-function opsins used by Muller-Ewald et al. [18], which may have influenced results. After 45 days withdrawal, LTD-like stimulation of vmPFC-NAshell projections increases incubated cocaine seeking while remodeling synapses, further indicating that this pathway can regulate cocaine seeking after abstinence [20]. When examined after 30 days of abstinence from the last self-administration session, pharmacological inactivation of the vmPFC attenuates, rather than increases, cue + context-primed relapse [15]. It is likely that the ability of pharmacological inactivation of the vmPFC to *attenuate* incubated cocaine seeking was due to off-target effects of baclofen and muscimol or to vmPFC projections to other brain regions. For example, the vmPFC also projects to the NA core [20,21] but little is known about how that projection regulates cocaine seeking. Taken together, this indicates that both the vmPFC and dmPFC are recruited by extinction training. However, dmPFC neurons containing NMDA receptors with NR2b-containing subunits may mediate relapse after abstinence without extinction.

The NA core is consistently found to be necessary for cued-cocaine seeking after both extinction and abstinence. The only study finding that it is not necessary for context-primed relapse after abstinence did not pair cocaine infusions with discrete cues during self-administration [25]. When rats are trained to self-administer cocaine in the presence of discrete cues, infusions of an mGlu5 receptor negative allosteric modulator (NAM) into the NA core attenuates context-primed relapse after abstinence [26]. These findings may have arisen due to the ability of discrete cocaine-paired cues to cause DA signaling in the NA core [32], thus recruiting this brain region into the circuitry mediating seeking during a relapse test. When rats are trained in the absence of discrete cues, the NA shell is necessary for context-primed reinstatement after extinction but not after abstinence [23,25]. This is consistent with the necessity of extinction to bring online the vmPFC cortex, a major efferent to the NA shell. When rats are trained with discrete cues, the NA shell is not necessary for cue-primed reinstatement [25]. However, deep brain stimulation of the NA shell attenuates cue-primed reinstatement, indicating a role for this brain region in mediating reinstatement [33]. Akin to the role of the vmPFC, the NA shell may be necessary for inhibiting, rather than driving, cue-primed reinstatement after extinction.

Inactivation of the dSTR (caudate/putamen) is uniformly necessary for cued cocaine seeking, regardless of whether extinction or abstinence occurred, prime (cue or context), and whether cocaine IVSA occurs in the presence of discrete cues [12,27,34,35]. Signaling through dSTR mGlu5 receptors is not necessary for context-primed relapse [26], consistent with evidence suggesting it is DA and not glutamate transmission in the dSTR that mediates such relapse [35]. Additional studies that did not investigate relapse per se find evidence that DA signaling in the dSTR mediates cue-induced cocaine seeking [36,37].

Regardless of whether rats are trained in IVSA in the presence of discrete cues, the vHipp and the BLA are necessary for reinstatement after extinction training when prompted by cues or context [10,13,14,28,29,30]. However, when rats are not trained in the presence of discrete cues, the BLA is not necessary for context-primed relapse after abstinence [12]. The dHipp is necessary for context-primed reinstatement after extinction, but not cue-primed [13]. Anatomical disconnection has demonstrated that the BLA-dHipp pathway is necessary for context-primed reinstatement after extinction [38]. Inactivation of the VTA is necessary for both cue-primed reinstatement and context-primed relapse after abstinence [25,31]. DREADD-mediated inhibition of rostral ventral pallidum-VTA projections were necessary for cue-primed reinstatement of cocaine seeking [39].

Taken together, a review of this literature reveals that extinction training is necessary to recruit the vmPFC and dmPFC regions of the PFC into the circuitry mediating cue/context-induced cocaine relapse. This is likely true for the NA shell as well. The dHipp is uniquely involved in processing the role of the drug-taking context, and not discrete cues. The dSTR, the NA core, and possibly the VTA are essential components of the drug seeking circuitry regardless of abstinence procedures, with the caveat that training rats to associate cues with IV cocaine is needed to bring the NA core online. This work highlights the role of discrete, drug-paired cues in recruiting circuitry mediating cocaine seeking. One major gap in knowledge is whether the results presented in Table 1 would change if female rodents were also included in such studies. As ovarian hormones have been found to influence cocaine-cue relationships [40], and one study finds a different pattern of cocaine cue-elicited brain activation in women with CUD [41], it is even more essential to assess the neurocircuitry of cue/context-induced cocaine seeking in female rodents. A number of regions have yet to be tested for their role in mediating incubation of cocaine seeking; however, several studies find strengthening of PFC-NA and BLA-NA pathways, and blockade of NA calcium-permeable AMPA (CP-AMPAs) in this model attenuates incubated cocaine-seeking [20,24,42,43]. Finally, there are additional studies that have used pathway specific strategies (e.g., anatomical disconnection or intersectional chemo/optogenetics) to further characterize projections mediating relapse. However, with the exception of the vmPFC-NA shell strategies discussed above, these approaches have not been used in both extinction and abstinence models and are thus not reviewed here.

Other rodent models utilizing punishment induced abstinence have been used, finding that reducing cocaine IVSA with punishment (foot shock) alters the neural circuitry underlying cue/context primed relapse. For example, when punishment was used to reduce cocaine-seeking, inactivation of the BLA increased context-primed relapse, whereas it attenuated context-primed relapse in rats experiencing extinction training without punishment [29]. Chemogenetic inhibition of the ventral pallidum attenuates cue- and context-primed relapse to cocaine-seeking in a punishment model, and this effect is most pronounced in the most punishment-resistant rats [44]. Thus, punishment induced abstinence also has the potential to alter the neurocircuitry underlying cocaine seeking. A voluntary choice-based abstinence model (e.g., choice between cocaine and food) has yet to be employed in the cocaine IVSA model and represents an important area to be explored.

## 3. Molecular Alterations Produced by Post-Cocaine Abstinence Regimens

In addition to remodeling the circuitry underlying cocaine relapse, behavioral experiences that follow cocaine IVSA can alter the molecular composition of synapses. Here, we summarize a large body of work aimed at understanding such changes in a brain region that underlies cocaine relapse after extinction and abstinence: the NA core. The examination of glutamate receptors, transporters, and proteins regulating their trafficking was chosen because of the established role of glutamate release in the NA core in driving cue-primed reinstatement after extinction [45] and both cue- and context-primed relapse following abstinence [46,47]. Within the NA core, antagonism of post-synaptic glutamate receptors attenuates cue-primed reinstatement of cocaine seeking (e.g., [24,26,48]). The NA core was also chosen because the comparison between the molecular consequences of extinction and abstinence has been explicitly made in a number of publications, providing sufficient data for us to compare and contrast between conditions. Such work has not been done in other parts of the relapse circuit.

Proteins of interest include subunits of the ionotropic AMPA (GluA1 and GluA2) and NMDA (GluN1 and GluN2a/b) receptors, as well as the metabotropic glutamate receptors (mGluRs), including mGlu1, mGlu5, and mGlu2. The glutamate transporter GLT-1 and the catalytic subunit of the cystine-glutamate exchanger, xCT, are important to assess, as they regulate glutamate homeostasis (balance between glutamate reuptake and release). Additionally, commonly assessed are proteins that regulate membrane trafficking, synaptic clustering, and signaling of these glutamate receptors: PSD-95, Narp and Homer 1b/c and Homer 2a/b. PSD-95 is a scaffolding protein that binds many receptors, channels, and signaling proteins and plays a key role in organization and function of the postsynaptic domain, predominately in excitatory synapses [49,50]. Narp is an extracellular protein of the pentraxin family co-regulating AMPA receptors clustering, and induction of long-term potentiation [51,52]. Homer 1b/c and 2a/b, so called “long-form” homers, are postsynaptic scaffolding proteins that regulate group I mGlu (mGlu1 and 5) signaling, such as signaling through IP3 receptors [53]. These proteins have been assessed in various parts of the synapse, including a whole tissue homogenate (H) in which a tissue punch is homogenized in a buffer and all parts retained. The S1 fraction is separated from the H by a low-speed centrifugation that removes the pellet (containing the cell nuclei and large debris), retaining the supernatant (S). A P2 “membrane-enriched” fraction is generated by high-speed centrifugation of the S1 fraction and retention and resuspension of the pellet. Thus, the P2 contains pre-synaptic, post-synaptic, and glial cell membranes. If the P2 resuspension is further spun at a high speed, the pellet generated will contain the LP1 fraction, containing a post-synaptic density (PSD) enriched fraction. The post-synaptic density is a protein-dense region attached to the post-synaptic membrane, containing synaptic scaffolding proteins. In addition, two techniques exist to isolate fractions containing only membrane proteins with significant cell surface presence, as achieved by labeling of cell-surface protein with membrane impermeable biotinylation reagent (also called surface biotinylation), or by irreversibly cross-linking of these proteins with the bis(sulphosuccinimidyl)suberate (BS3) agent.

**Figure 1:**
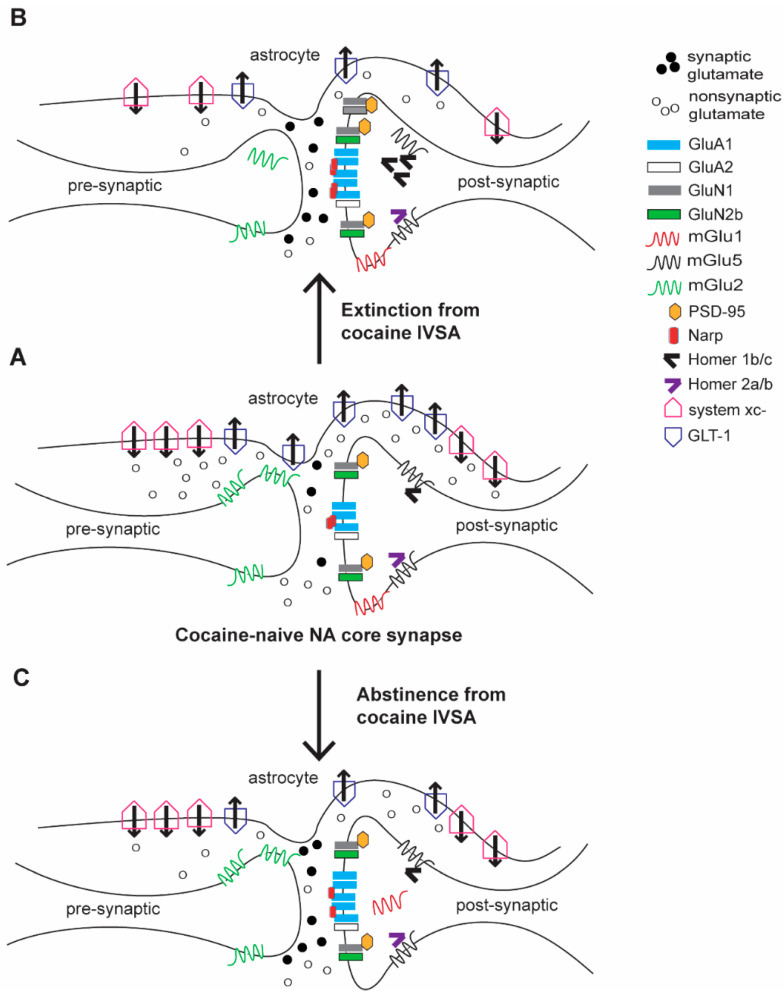
Molecular adaptations in glutamate receptors, transporters, and signaling molecules in the NA core synapse after extinction and abstinence from cocaine. (**A**) Prototypical glutamate synapse in a cocaine-naïve animal. (**B**) Adaptations after extinction training differ from those after abstinence (**C**), with different effects on GluN1, mGlu5, mGlu1, and several scaffolding proteins.

Regardless of whether extinction or abstinence occurred, surface expression of GluA1 is consistently increased in the NA core [24,54,55]. Accompanying changes in total or PSD protein expression are not always found, with the exception of one study finding that GluA1 is increased in the S1 fraction [24] and one finding increased expression in the LP1 fraction [55]. Surface and total GluA2 expression is consistently found to be unchanged [24,54,55]. There is some evidence for its increased presence in the PSD fraction after both extinction and abstinence [56], which could indicate a readily available pool for insertion into the membrane or recently removed protein. The presence of increased surface GluA1 in the absence of increased Glu2 indicates the presence of calcium-permeable (CP) AMPA receptors, also referred to as “GluA2-lacking AMPAs”. Such receptors are high-conductance and are not typically found in drug-naïve rodents [57]. Presence of these receptors has been confirmed with 1-naphthylacetylsperimine (Naspm), a specific antagonist of CP-AMPAs [24,58], which reduces EPSCs and/or reinstatement of cocaine-seeking. Furthermore, CP-AMPAs are found in the NA after abstinence from extended- (6 h/d) but not short-access (2 h/d) cocaine IVSA [59]. However, when extinction training is used following 2 h/d cocaine self-administration, there is evidence of CP-AMPA formation: intra-NA infusion of Naspm attenuates reinstatement and increased surface GluA1 is found [54,58]. It should be noted that these changes are also observed in NA shell alone [24] and when samples include both NA core and shell tissue [55,60]. The significance of CP-AMPA formation for cocaine seeking is evidenced by consistent findings that manipulations to inhibit CP-AMPAs with Naspm or reduce the formation of CP-AMPAs with an mGlu1 positive allosteric modulator (PAM) attenuate cocaine-seeking. See Figure 1 for subcellular location of these proteins (A) and their changes after extinction (B) and abstinence (C).

The GluN1 subunit of the NMDA receptor has been found to be increased in H and S1 fractions after extinction, but not abstinence [61,62,63]. Thus, this represents one protein that is upregulated by extinction training. Interestingly, in post-mortem tissue from cocaine overdose victims, the GluN1 subunit is also upregulated [64]. However, those victims had recently ingested cocaine, and at this time it is not known if such increases would persist after a drug-free period.

At 21 days post-IVSA, surface mGlu5 is only decreased following extinction training, and not abstinence [56]. However, when examined after longer periods of abstinence (48 days), there is a modest decrease in surface mGlu5 [60]. mGlu5 is consistently found to be unchanged in the total protein fraction (H and S1), with the exception of a decrease observed in females, and when only the monomer band of this receptor was quantified (the dimerized form is usually the active form in the membrane) [56,65,66]. mGlu5 has been observed to be increased in the PSD fraction after extinction, combined with increased expression of its scaffolding protein Homer 1b/c, and a decrease in surface expression [56]. Thus, Homer 1b/c likely is upregulated by extinction, which internalizes mGlu5, resulting in its retention in the PSD fraction. This Homer 1b/c upregulation may be protective against relapse, as AAV-mediated upregulation of NA core Homer 1b/c attenuates cue-primed reinstatement [56]. However, when decreased surface mGlu5 was observed at 48 days of abstinence, this was not accompanied by increased Homer 1b/c or Homer 2a/b [60]. Similarly, while increased mGlu5 was observed in the PSD fraction after 14 days of abstinence, it was not accompanied by increased Homer 1b/c [65]. Thus, the decrease in surface mGlu5 after extinction and abstinence may be mediated by different trafficking mechanisms. Note that there were slight differences in the fractionation protocol in these two studies assessing mGlu5 and Homer 1b/c in the “PSD-enriched fraction”. This fraction contained synaptophysin in one study [65] whereas it did not in the second study [56]. Furthermore, 10 days [65] of abstinence was used in one study and 21 d in the other [56]. Two additional scaffolding proteins, Narp and PSD-95 were also upregulated by extinction training, which potentially contributes to accompanying changes in plasticity, such as the loss of the ability to induce LTP after extinction training [56]. Homer 2a/b has been assessed only after abstinence, with both a decrease and no change observed. The reason for this discrepancy is not clear, as both studies utilized extended access (6 h/day) IVSA and assessed expression at 14 days of withdrawal in the in the S1 fraction [60,67].

Relative to mGlu5, mGlu1 has been examined in few studies. One found no change in mGlu1 expression in the PSD fraction after 21 days of abstinence or extinction [56]. However, after 48 days of abstinence, total and surface expression of mGlu1 was decreased, while surface expression was also decreased after 25 days of abstinence [60]. This decrease was found to enable CP-AMPA formation. The Group II mGlu receptors mGlu2 and mGlu3 have only recently been able to be distinguished by antibodies due to their structural similarity. After 14 days of extinction from cocaine IVSA, mGlu3 expression was unaffected, but mGlu2 expression was reduced in both the total and surface fractions.

GLT-1 and xCT are proteins that regulate glutamate homeostasis, the balance between glutamate reuptake and release. GLT-1 is the major glutamate transporter, accounting for at least 90% of glutamate reuptake [68]. The protein xCT is the catalytic subunit of system xc^-^, which exchanges extracellular cystine for intracellular glutamate. System xc^-^ is the main source of basal, nonsynaptic glutamate release in the NA core [69]. A number of studies have reported GLT-1 to be decreased in total protein, P2, and surface fractions after extinction from cocaine IVSA [5,54,66,70,71,72]. Only one has examined expression after abstinence, finding a decrease in the total protein fraction [73]. Similarly, xCT has consistently been found to be decreased in total and P2 fractions after extinction [54,66,70,71], but has not yet been examined after abstinence. As basal glutamate levels are decreased following abstinence [74], and such decreases have been directly linked to system xc^-^ function [75], it would be expected that xCT would be decreased after abstinence. Both xCT and GLT-1 are decreased after extinction in female rats [66].

As summarized in Table 2, the most consistent molecular consequences of extinction training are increased GluN1 and an early decrease in surface mGlu5 expression that is accompanied by increased expression of the associated protein Homer 1b/c. Overall, decreased mGlu5 is only observed in the NA core under certain conditions. It is decreased after 14–23 days of extinction training only in the surface fraction in males, but in the total protein fraction in females. Following abstinence without extinction, surface mGlu5 is decreased only after long periods of abstinence (i.e., only at 48 days of withdrawal). Importantly, two studies in humans with CUD using the mGlu5 PET ligand [^11^C]ABP688 find decreased mGlu5 availability in the striatum [76,77]. This supports the idea that when studying the ability of mGlu5-targeting ligands to attenuate cocaine seeking, either extinction training or longer periods of abstinence (~48 days) need to be employed.

## 4. Differences in Relapse Attenuation by Pharmacological Strategies Following Different Post-Cocaine Regimens

Due to reported effects of post-cocaine regimen on glutamatergic protein expression, many drugs targeting the glutamate system have been tested for their ability to attenuate relapse after both extinction training and abstinence. For example, the mGlu5 NAM MTEP attenuates context-primed relapse after abstinence as well as cue-primed relapse after extinction both when given systemically and when infused into the NA core [26,48,79]. Naspm, the specific antagonist of GluA2-lacking AMPA receptors, attenuates incubated cocaine-seeking after abstinence when infused into the NA [24]. Similarly, Naspm attenuates cocaine-primed reinstatement after extinction [58]. While it has not yet been tested for cue-primed reinstatement after extinction, it would likely be effective, as the finding that GluA2-lacking receptors form after extinction would possibly render the medium spiny neurons responsive to Naspm during a cue-primed test.

Two drugs that target glutamate homeostasis, the antibiotic ceftriaxone and N-acetylcysteine, have been tested in both extinction and abstinence models. Ceftriaxone attenuates cue-primed reinstatement after extinction and abstinence and context-primed relapse after abstinence [46,47,70]. Ceftriaxone prevents the glutamate efflux that drives relapse when it is administered for 5–7 days during extinction training preceding a cue + cocaine or cocaine-primed reinstatement test and when administered during the 6 days of abstinence preceding a cue + context primed relapse test [5,47,80]. However, the ability of ceftriaxone to attenuate such glutamate efflux is not as robust during a context primed relapse test after abstinence, indicating that ceftriaxone interacts with the circuitry mediating context-primed relapse to a lesser degree than with circuitry mediating cocaine- and cue-primed relapse [46]. Similarly, while subchronic administration of a low dose of *N*-acetylcysteine (60 mg/kg) attenuates cue-primed reinstatement after extinction, it does not reduce context-primed relapse after abstinence [81]. Thus, cue-primed reinstatement after abstinence engages a neurocircuitry distinct from that engaged during context-primed relapse.

Ceftriaxone has also been tested for its ability to attenuate cued relapse after Pavlovian extinction (cue extinction therapy) of discrete cocaine-paired cues. In this study, the Pavlovian extinction sessions occurred in a context distinct from the self-administration context (e.g., different operant boxes with distinct floors and odors). The combination of ceftriaxone and cue extinction failed to additively or synergistically reduce cued relapse. This is consistent with two randomized controlled studies in humans with CUD showing that another compound which targets the glutamate system, D-cycloserine, had no effect or a worsening effect on cocaine cue reactivity [82,83]. It should be noted that in rodents, when cue extinction is conducted in the same context as cocaine self-administration, cue-primed relapse is attenuated [84]. The same study found that when D-cycloserine is administered immediately after a cue extinction session conducted in an alternate context, cued relapse is attenuated in the cocaine self-administration context [84]. Taken together, while both D-cycloserine and ceftriaxone target the glutamate system, only the former is able to render cue extinction therapy successful when it is conducted outside the cocaine-taking environment. Unfortunately, those positive effects do not translate into humans.

## 5. Conclusions

These results indicate that learning about cue-cocaine relationships and extinguishing the instrumental response made to obtain drug alter the neurobiology observed in late abstinence from cocaine. Extinction training recruits PFC regions to mediate later relapse. However, these conclusions are based on the work of inactivation studies, and at least one study administering specific GluN2b antagonist into the dmPFC finds a role for this brain region in mediating relapse after abstinence. Future work using more pathway-specific manipulations may find a role for this brain region in mediating relapse after abstinence. In support of this idea, the PFC-NA core pathway is strengthened in abstinence [43] and glutamate transmission in the NA core is necessary for incubated cocaine seeking. The dHipp is only involved in context-primed and not cue-primed relapse. The dSTR and the NA core are important for relapse after both abstinence and extinction; however, training rats to self-administer using discrete cocaine-paired cues is necessary to bring the NA core into the circuitry mediating relapse after abstinence [26].

There are also differences in expression of the NMDA receptor subunit GluN1 and in surface expression of mGlu5 after extinction compared to abstinence. The differences in mGlu5 expression do not lead to an inability of intra-NA core MTEP to attenuate reinstatement of cocaine seeking after extinction [26]. This is likely because MTEP is a negative allosteric modulator and having less mGlu5 available to bind after extinction training would by itself reduce reinstatement. Alternatively, it is possible that this reduction in surface mGlu5 would lead to the inability of mGlu5 PAMs, such as CDPPB, from attenuating relapse after extinction. Such PAMs are more attractive therapeutics for CUD because they enhance learning, while NAMs carry the risk of learning impairments [85]. At this time, only one mGlu5 PAM has been tested for its ability to reduce relapse and this was done in an abstinence model, finding that it attenuates cue+context-primed relapse after 45-60 days of abstinence [85]. The consequences of GluN1 upregulation after extinction training are currently unknown but may be one of the players in the ability of glutamate homeostasis targeting compounds (ceftriaxone and N-acetylcysteine) to better attenuate cued-cocaine seeking after extinction.

What about the construct and criterion validity of extinction and abstinence models? Regarding the protein expression data presented here, only mGlu5 can be visualized in the human brain with the use of radioligands and PET. Akin to what is observed in rodents, striatal mGlu5 availability is decreased in humans with CUD. GluN1 expression is increased following extinction from cocaine in rodents and in human cocaine overdose victims [64]. In terms of the neural circuitry mediating relapse, some evidence exists for decreased function of the human ventromedial PFC in CUD, which along with decision-making capabilities, recovers over the length of abstinence [86,87]. While cocaine-related cues produce increased activity in both dorsal and ventral regions of the PFC in those with CUD [88], subjects in such studies are not known to be in abstinence, and the length of time since last cocaine use is an important variable in such studies. Regarding the validity of pharmacotherapeutics to reduce cocaine use in CUD, of the compounds discussed here, only N-acetylcysteine has been tested in humans. N-acetylcysteine reduced cocaine craving and cocaine-cue reactivity in a pilot study [89]. A double-blind, placebo-controlled trial found that it was only able to reduce craving and lengthen the time to relapse in those participants who had achieved abstinence at the start of the trial [90]. This is consistent with work in rats showing that *N*-acetylcysteine does not reduce ongoing self-administration [91] and further supports the idea that post-cocaine abstinence alters neurocircuitry underlying cocaine seeking. Taken together, the results from humans are in agreement with findings from studies in rodents employing extinction training following cocaine IVSA. This is an important conclusion; especially as instrumental extinction does not have face validity for the experience of cocaine users.

The field could be advanced by a better understanding of what individuals with CUD experience during drug abstinence. This involves characterizing the context of drug-taking and abstinence. While it has widely been assumed that humans do not undergo abstinence in the same context in which drug-taking occurred, our recent interviews with cocaine users reveal that many use cocaine in their personal automobiles and places of residence. This would indicate that a population of users extinguish their drug-taking context. Whether the discrete drug-paired cues (e.g., paraphernalia) are also being extinguished is unclear. However, others have found that use occurs outside the home [92]. Pure abstinence outside the drug-taking context could map onto the situation in which a user is incarcerated or is in a rehabilitation center. Voluntary abstinence would likely only apply to a sub-population of users undergoing contingency management. In conclusion, because different neuroadaptations arise from the use of different post-cocaine regimens in rodents, the use of multiple methods will allow the characterization of brain changes encompassing the wide range of circumstances experienced by individuals with CUD. It remains important to be aware of the ways in which extinction and abstinence interact with drug-paired contexts and discrete cues to impact dependent variables of interest of rodent studies.

## Figures and Tables

**Table 1:** Neurocircuitry of cocaine relapse after abstinence or extinction. Arrows indicate the effect of inactivation of this brain region (using pharmacological, chemogenetic, or optogenetic strategies) on relapse. Effects of stimulation are also noted for the vmPFC. * indicates that instead of inactivation strategies, glutamate receptor antagonism was used. Grey text is used to indicate studies in which discrete cues were not paired with intravenous cocaine delivery.

Brain Region	Extinction		Abstinence	
	Cue-Primed Reinstatement	Context-Primed Reinstatement	Cue + Context-Primed Relapse	Context-Primed Relapse
**dmPFC**	↓ [10]	↓ [13]	↔ [15]	↔ [12]
			↓ * [16]	
**vmPFC**	↔ [10]	↔ [13]	↓ [15]	
	↓ by stimulation [17,18]	↑ [22]	↔ [17,18]	
**NA core**	↓ [11]	↓ [23]	↓ * [24]	↔ [25]
				↓ * [26]
**NA shell**	↔ [11]	↓ [23]		↔ [25]
**d.STR**	↓ [12]	↓ [12]		↓ [12,25,27]
				↓ * [26]
**BLA**	↓ [10,28]	↓ [13,29]		↔ [12]
**dHipp**	↔ [13]	↓ [13]		
**vHipp**	↓ [14]	↓ [30]		
**VTA**	↓ * [31]			↓ [25]

**Table 2:** NA core protein changes after ≥10 days of extinction or abstinence from cocaine self-administration. H= Whole tissue homogenate. S1 = total protein fraction without nuclear material. P2 = membrane-enriched fraction. LP1 = post-synaptic density-enriched fraction. Surface = surface membrane proteins.

Protein	Extinction (vs. Drug-Naïve Control)			Abstinence (vs. Drug-Naïve Control)		
	Change	Fraction	Citation(s)	Change	Fraction	Citation(s)
**GluA1**	↔	H	[62]	↔	H	[62]
	↔	S1	[54]	↑	S1	[24]
	↔, ↑	LP1	[56,62]	↔	LP1	[56,62]
	↑	Surface	[54]	↑	Surface	[24,55,59]
**GluA2**	↔	S1	[54]	↔	S1	[24]
	↔	LP1	[56]	↔	LP1	[55,56]
	↔	Surface	[54]	↔	Surface	[24,55,59]
**GluN1**	↔ (6 h IVSA)	H	[62]	↔	H	[62]
	↑ (2 h IVSA)		[61]			
	↑	S1	[63]			
	↔	LP1	[62]	↔	LP1	[62]
**mGlu1**				↓	S1	[60]
	↔	LP1	[56]	↔	LP1	[56]
				↓	Surface	[60]
**mGlu5**	↔	H	[65]	↔	H	
	↔, ↓ (in females)	S1	[56,66]	↔, ↔, ↓	S1	[56,60,67]
	↑, ↔	LP1	[56,65]	↔, ↑	LP1	[56,65]
	↓	Surface	[56]	↔, ↓ (only at 48 d)	Surface	[56,60]
**mGlu2**	↓	S1	[78]			
	↓	Surface	[78]			
**xCT**	↓	S1	[54,66,71]			
	↓	P2	[70]			
**GLT-1**	↓	S1	[54,66,71]	↓	S1	[73]
	↓	P2	[70,72]			
	↓	Surface	[5]			
**PSD-95**	↑	LP1	[56]	↔	LP1	[56]
**Narp**	↑	LP1	[56]	↔	LP1	[56]
**Homer 1b/c**	↔	H	[65]	↔	H	[65]
	↔	S1	[63]	↔	S1	[60,67]
	**↑, ↔**	LP1	**[65]**	**↔**	LP1	**[65]**
